# Cell type-specific pharmacological modulation of the neuro-immune axis: the role of ADRB2 signaling in reshaping the tumor ecosystem

**DOI:** 10.3389/fphar.2026.1848277

**Published:** 2026-05-28

**Authors:** Wenjun Meng, Mengting Li, Ruoyu Ren, Xiaoli Mu, Bin Hou, Qing Li

**Affiliations:** 1 Department of Pain Management, West China Hospital, Sichuan University, Chengdu, China; 2 Cancer Center, West China Hospital, Sichuan University, Chengdu, China; 3 Hemodialysis Unit, West China Tianfu Hospital, Sichuan University, Chengdu, China; 4 West China School of Medicine, Sichuan University, Chengdu, China; 5 Health Management Center, General Practice Medical Center, West China Hospital, Sichuan University, Chengdu, China; 6 Department of Rehabilitation Medicine, West China School of Public Health and West China Fourth Hospital, Sichuan University, Chengdu, China; 7 Department of Biotherapy, Cancer Center, West China Hospital, Sichuan University, Chengdu, China; 8 Department of Biotherapy, Cancer Center, National Clinical Research Center for Geriatrics, West China Hospital, Sichuan University, Chengdu, China

**Keywords:** ADRB2, cell type-specific targeting, immune modulation, neuro-immune axis, tumor microenvironment, β-adrenergic signaling, β-blocker

## Abstract

Adrenergic signaling through β2-adrenergic receptor (ADRB2) is a conserved neuro-immune axis that modulates tumor biology at multiple levels: tumor cells, stromal compartments (CAF, endothelium), myeloid populations (MDSC/TAM), and lymphocytes (CD4^+^/CD8^+^). Activation of ADRB2 by stress-related catecholamines (norepinephrine/epinephrine) promotes angiogenesis, pro-metastatic programs and immunosuppression; conversely, ADRB2 blockade in preclinical models reprograms myeloid cells, potentiates T cell-mediated antitumor immunity and synergizes with immune checkpoint blockade in defined contexts. Clinical and epidemiologic data remained heterogeneous, but other small randomized and retrospective studies suggested that short-term pharmacologic suppression of adrenergic signaling (often combined with COX-2 inhibition) can favorably affect biomarkers and, in pilot trials, disease-free survival. This review summarized cell type-specific mechanisms linking ADRB2 to tumor ecosystem remodeling, compared pharmacological strategies for selective and cell-directed ADRB2 modulation, and outlined translational priorities and trial designs to move ADRB2-targeted approaches into practice.

## 1 Introduction

Tumor is no longer considered as a purely cell-autonomous disease, because their occurrence and development are highly dependent on dynamic interactions with the host microenvironment (Jiang et al., 2020). The tumor microenvironment (TME) is a complex ecosystem that includes immune cells, fibroblasts, vascular endothelial cells, and nerve fibers, which have received much attention in recent years (de V et al., 2023; Meng et al., 2024; Kobayashi et al., 2025; Meng et al., 2025a). The sympathetic nervous system (SNS) is the major source of catecholamines in most tumor settings; however, non-neuronal cells may also contribute to local catecholamine pools in selected contexts, thereby enabling autocrine/paracrine adrenergic signaling within the TME (Xiao et al., 2023;Lauten et al., 2025). The SNS, by releasing these neurotransmitters (such as norepinephrine, NE), binds to adrenergic receptors on the surface of immune cells and tumor cells, forming a “neuro-immune axis” that impacts tumor progression (Lyu et al., 2024; Jin et al., 2026; Wang et al., 2026; Vanden Abeele and Salzet, 2025; Vermeer et al., 2025; Shao et al., 2026; Zhang S. et al., 2026). NE signals *via* β-adrenergic receptors (β-AR, e.g., ADRB1/2/3), with ADRB2 being broadly expressed across tumor cells, endothelial cells, and multiple immune subsets, where it serves as a central mediator of stress-associated tumor biology (Fjæstad et al., 2025). But the biological consequences of the signaling of ADRB2 are highly cell-type specific (Kobayashi et al., 2025).

Seminal mechanistic studies demonstrated that chronic adrenergic stimulation enhances angiogenesis, matrix remodeling and metastatic dissemination through the cAMP-PKA axis and downstream effectors (VEGF, MMPs, HIF-1α) in tumor cells (Thaker et al., 2006). Moreover, adrenergic signals can rewire immune cell metabolism and differentiation, favor myeloid immunosuppressive phenotypes, and impair effector T Cell responses, thereby undermine anti-tumor immunity and responsiveness to immunotherapy (Wang et al., 2025). Recent single-cell and spatial transcriptomic analyses have also started to map ADRB2 expression across TME compartments, revealing patterns of cell-type-specific vulnerability that present opportunities for selective pharmacological intervention (Meng X. L. et al., 2026; Kim et al., 2026).

Repurposing non-selective β-blockers (e.g., propranolol) and developing selective β2 antagonists, biased ligands, or targeted delivery systems may allow modulation of ADRB2 signaling to reshape the tumor ecosystem while limiting systemic cardiovascular and tissue toxicity (Chung et al., 2016). Clinical studies in colorectal cancer provided a proof-of-concept that short, timed adrenergic blockade can favorably alter biomarkers and possibly clinical outcomes in select contexts, but evidence remains mixed and heterogeneous across tumor types and study designs (Ricon-Becker et al., 2023; Wang et al., 2022; Gleber-Netto et al., 2026).

Therefore, this review synthesized cell-type-specific mechanistic evidence for ADRB2’s role in TME remodeling, compare pharmacological strategies, and propose focused translational pathways and trial designs to test ADRB2-centered therapies.

## 2 The role of the ADRB2 signaling pathway in the neuro-immune axis of tumors

### 2.1 The sympathetic nervous system and tumor innervation

The sympathetic neurotransmitter NE promotes the malignant progression of various tumors by activating ADRB2 located on the surface of tumor cells. For instance, in triple-negative breast cancer, the NE/ADRB2 signaling pathway not only stimulates cancer cell proliferation but also induces the secretion of nerve growth factor, thereby fostering sympathetic nerve sprouting and establishing a vicious cycle (Jin et al., 2023). In neurilemmoma of the peripheral nerve, epinephrine stimulates ADRB2 to activate the Hippo signaling transducers YAP/TAZ, thus expanding the population of cancer stem-like cells and exacerbating the tumor malignancy (Huang et al., 2021). This neurotransmitter-driven signaling not only directly impacts tumor cells but also reshapes the TME. For example, in ovarian cancer models, chronic stress leads to stress hormones elevating; by activating relevant receptors (including ADRB2), these hormones promote the infiltration of immunosuppressive myeloid-derived suppressor cells into the TME, then drive tumor progression (Rivera-López et al., 2025). Collectively, these findings underscore the SNS’s pivotal role in acting through receptors, such as ADRB2, in the bidirectional regulation of both tumor cells and the tumor immune microenvironment (TIME) in which they reside.

### 2.2 Structure, distribution and signal transduction mechanism of ADRB2

ADRB2 is a member of the G protein-coupled receptor (GPCR) superfamily, the largest family of cell-surface membrane receptors characterized by seven transmembrane α-helical domains and broad responsiveness to diverse extracellular cues; its structural features underpin its extensive signal transduction capabilities (Zhang et al., 2024; Meng et al., 2025b). After ADRB2’s activation, it mainly transmits signals through the classical Gs-adenylate cyclase-cAMP-PKA-CREB signaling pathway (Kuroda and Higashi, 2021). This pathway can regulate the transcription of a variety of ADRB2’s downstream genes. For example, ADRB2 activation can enhance the transcription of hyaluronic acid synthase 2 (HAS2), thus increasing the production of hyaluronic acid, an extracellular matrix component (Kuroda and Higashi, 2021). This process plays an important role in cancer progression and wound healing. Apart from the classical pathway, ADRB2 can also interact with other oncogenic pathways. In head and neck squamous cell carcinoma (HNSCC), ADRB2 signaling interacts with common tyrosine kinase receptor pathways such as MAPK and PI3K; its blockade can not only inhibit the p38 and NF-κB oncogenic pathways, but also strongly affect the ERK and PI3K pathways (Mele et al., 2020). Additionally, ADRB2 can form multimeric complexes with other GPCRs. For example, in breast cancer cells and lung cancer cells, ADRB2 forms heteromeric complexes with CXCR4. This interaction is regulated by ligands and significantly affects the multimerization state of the receptor, suggesting the complexity of its signal transduction in tumor cells (Liang et al., 2024). Polymorphisms of the ADRB2 gene have been shown to be related to its transcriptional activity, mRNA translation, receptor expression and sensitivity, which affect cancer susceptibility, patient prognosis and treatment response (Wang and Jiang, 2021).

### 2.3 Functional similarities and differences between ADRB2 and other adrenergic receptors in the TME

In the TME, members of the adrenergic receptor family (such as α and β receptors) collectively receive neural signals; however, ADRB2 exhibits unique and extensive functions, particularly in bridging neural signaling with immunosuppression (Wang and Jiang, 2021). Compared to other adrenergic receptors, ADRB2 plays an indispensable role in regulating the immunosuppressive functions of myeloid-derived suppressor cells (MDSCs). During tumor growth, ADRB2 expression on MDSCs increases; its signaling pathway operates by altering MDSC metabolism (specifically by reducing glycolysis while increasing oxidative phosphorylation and fatty acid oxidation, and by inducing autophagy and activating the arachidonic acid cascade) (Mohammadpour et al., 2021). Ultimately, this process drives the release of the immunosuppressive mediator PGE2, thereby enhancing the immunosuppressive activity of MDSCs. This precise regulation of specific immune cell subsets constitutes a salient feature of ADRB2 function. Within tumor cells themselves, ADRB2 signaling also manifests unique pro-tumorigenic mechanisms. For instance, in lung adenocarcinoma, the upregulation of ADRB2’s expression promotes cancer cell proliferation, migration, and invasion by activating the JAK2/STAT3 signaling pathway (Xu et al., 2025). Conversely, network pharmacology analyses of gastric cancer have identified ADRB2 alongside genes such as CAV1, as a key target with potential anti-gastric cancer utility, suggesting that its signaling pathway may serve as a critical node for therapeutic intervention (Fatima et al., 2024). Collectively, these evidence demonstrated that ADRB2 acts not merely as a key effector of sympathetic nervous signaling, but rather as a central hub within the TME that interconnects neural innervation, cancer cell behavior, and the state of immunosuppression; consequently, its functional scope is far more extensive and profound than that of other adrenergic receptors.

## 3 Cell type-specific functions of ADRB2 signaling in key TME components

### 3.1 Tumor cells: promoting malignant progression

#### 3.1.1 Proliferation, survival, and maintenance of stemness

Adrenergic signaling, such as NE treatment, can significantly impact the biological behavior of triple-negative breast cancer cell lines (Carrasco et al., 2025). Although NE was observed to reduce spheroid size in 3D spheroid models, this finding may reflect a complex regulatory effect on cell adhesion or invasion rather than mere inhibition of proliferation. Proteomic analysis has revealed that basal-like breast cancer cells exhibit a significant response to adrenergic signaling, involving the reprogramming of pathways related to proliferation, cytoskeletal dynamics, and metabolism (Carrasco et al., 2025). In lung cancer, ADRB2 is identified as one of the prognostic genes associated with non-apoptotic forms of regulated cell death, suggesting that it may play a role in regulating tumor cell survival (Li et al., 2023).

#### 3.1.2 Epithelial-mesenchymal (EMT) transition, invasion, and metastasis

In gastric cancer, chronic stress activates ADRB2 and the glucocorticoid receptor (GR), thereby initiating downstream pathways such as cAMP/PKA and NF-κB/STAT3; ultimately, this enhances the EMT process, upregulates the expression of matrix metalloproteinases (MMPs), promotes angiogenesis, then accelerating tumor metastasis (Chen et al., 2025). In breast cancer cells, NE treatment can reduce the expression of EMT-related markers; notably, this effect can be partially reversed by the β-AR antagonist propranolol, providing direct evidence of the central role of ADRB2 signaling in regulating EMT (Carrasco et al., 2025). In models of colorectal cancer liver metastasis, NE utilizes ADRB2 signaling to reprogram Kupffer cells, inducing them to secrete CXCL12, which in turn promotes the migration of colorectal cancer cells (Li et al., 2026). It reveals a novel mechanism by which ADRB2 signaling facilitates metastasis through indirect tumor-stroma interactions. Thus, ADRB2 signaling is a key factor driving EMT in tumor cells and enhancing their invasive and metastatic capabilities.

#### 3.1.3 Metabolic reprogramming

Chronic stress is closely associated with alterations in tumor metabolism (Trumpff et al., 2024; Tian et al., 2021). In gastric cancer, stress-driven epigenetic and metabolic reprogramming amplifies the Warburg effect (aerobic glycolysis), thereby providing tumor cells with the energy and biosynthetic precursors necessary for malignant progression (Chen et al., 2025). Although specific metabolic enzymes or pathways have not been explicitly linked to ADRB2 in current studies, given the canonical role of adrenergic signaling in systemic metabolic regulation, as well as its extensive influence on various cell types within the TME, it is highly probable that ADRB2 signaling participates, either directly or indirectly, in shaping a metabolic microenvironment conducive to tumor growth.

### 3.2 Stromal cells: constructing supportive niches

#### 3.2.1 Activation of cancer-associated fibroblasts (CAFs) and extracellular matrix (ECM)

CAFs constitute a major component of the tumor stroma, and their activation status is critical for establishing a niche that supports tumor growth and metastasis (Liu et al., 2022). Currently, a lack of studies on directly addressing the regulation of CAFs by ADRB2, but the overarching framework of the neuro-immune-stromal axis lends support to this concept (Zhang F. et al., 2026; Yuan et al., 2025). NE, released primarily by sympathetic nerves, can act upon ADRB2 receptors on fibroblasts, thereby inducing their conversion into an activated CAF phenotype. Activated CAFs are capable of secreting copious amounts of ECM components, growth factors, perineural invasion (PNI), and inflammatory cytokines, thereby promoting tumor proliferation, invasion, and angiogenesis, while simultaneously suppressing immune responses (Li et al., 2025; Meng W. et al., 2026). CAFs may also produce factors sustaining local neural growth, establishing a reciprocal loop (Lyu et al., 2024; Xue et al., 2023). ADRB2 expression in stromal elements influences the balance between tumor-promoting vs. tumor-restraining CAF subtypes. Consequently, targeting ADRB2 signaling may inhibit CAF activation, thereby disrupting the supportive tumor stroma. In the future, selective targeting of stromal ADRB2 signaling (e.g., CAF-directed delivery) could reduce pro-invasive niches without systemic β-blockade toxicity. In this review, CAFs are depicted as ADRB2-responsive stromal target cells rather than established NE-producing cells (Eng et al., 2014; Hanoun et al., 2015).

#### 3.2.2 Endothelial cells and vasculature

Here, endothelial cells are also presented as ADRB2-responsive target cells downstream of sympathetic signaling, rather than as canonical NE-secreting cells (Eng et al., 2014; Hanoun et al., 2015). Sympathetic innervation remodels the metabolic state and vascular phenotype of endothelial cells, with the ADRB2 signaling pathway playing a central role in this process. ADRB2 activation drives pro-angiogenic metabolic reprogramming (characterized by enhanced glycolysis) and increases vascular permeability; both alterations collectively influence immune cell infiltration and hypoxia in the TME (18, 20). Mechanistically, ADRB2 signaling can directly act on vascular endothelial cells, promoting their proliferation, migration, and lumen formation, or it can promote angiogenesis by activating downstream pathways such as NF-κB/STAT3, thereby driving tumor progression and metastasis (Chen et al., 2025). Furthermore, ADRB2 can indirectly enhance angiogenesis by remodeling the secretory profile of tumor cells and stromal cells (such as TAMs and CAFs), for example, by inducing the expression of pro-angiogenic factors such as VEGF (Li et al., 2026; Kilpatrick et al., 2019). Correspondingly, blocking adrenergic signaling has been shown to inhibit angiogenesis and promote vascular normalization, thereby improving immune cell infiltration (Wang et al., 2025; Kim et al., 2026). Therefore, combining ADRB2 modulation with agents that rely on immune access (e.g., checkpoint inhibitors, adoptive T Cell therapies) may be synergistic if vascular normalization is achieved.

#### 3.2.3 Myeloid compartment (MDSC/TAM)

The ADRB2 signaling pathway may also influence the recruitment and function of MDSCs. Chronic psychological stress promotes the expansion and infiltration of MDSCs, particularly polymorphonuclear-MDSCs, via stress hormone-mediated activation of glucocorticoid receptors (GR) and ADRB2-dependent pathways (e.g., Notch), thereby enhancing immunosuppression and tumor progression (Rivera-López et al., 2025). β-blockade can reduce the monocyte/MDSC burden and reprogram residual myeloid cells to an antigen-presenting phenotype with higher MHC-II/CD86 expression, enabling CD4^+^ Th1 responses in models (Shin et al., 2026). Temporal analyses in murine metastasis models showed monocyte reduction precedes Th1 CD4^+^ increases, suggesting causality (Fjæstad et al., 2025). This is a high-value target because myeloid cells are dominant mediators of immune suppression across tumor types, highlighting ADRB2 blockade as a potential strategy to limit MDSC accumulation and function.

The ADRB2 signaling pathway drives tumor-associated macrophages (TAMs) toward immunosuppressive M2 phenotype polarization. NE activates the PI3K/Akt signaling pathway by acting on ADRB2 on Kupffer cells, thereby promoting their polarization toward M2-like phenotype (Li et al., 2026). Similarly, in gastric cancer, chronic stress activates the SNS to release catecholamines, which act on cells in the TME and induce TAMs toward M2 polarization, which is one of the important mechanisms for the formation of an immunosuppressive microenvironment (Chen et al., 2025). This suggests that targeting ADRB2 signaling may be an effective strategy to reverse TAM function and improve immunosuppressive status.

#### 3.2.4 T lymphocytes: suppression and depletion of cytotoxic function

ADRB2 signaling exerts a predominantly suppressive effect on T lymphocyte function, particularly in cytotoxic CD8^+^ T cells, and represents a key neuro-immune axis driving tumor immune evasion (Chen et al., 2025). Chronic stress-induced adrenergic signaling has been linked to CD8^+^ T cell exhaustion and impaired effector function, partly through disruption of metabolic reprogramming during activation, resulting in reduced IFNγ and granzyme production as well as diminished responsiveness to immune checkpoint blockade (ICB) (Wang et al., 2025; Pu et al., 2025). Consistently, dysregulation of the neuro-immune axis is considered a central mechanism shaping the immunosuppressive TME and contributing to therapeutic resistance (Zhang F. et al., 2026). Mechanistically, ADRB2 signaling can both directly impair T cell function and indirectly reinforce immunosuppression *via* myeloid and stromal interactions (Zhang F. et al., 2026). Although β-adrenergic stimulation may transiently enhance CD8^+^ T cell recruitment and activation in specific contexts (such as CXCL10 induction in p53-mutant HNSCC), this effect is accompanied by upregulation of exhaustion markers, indicating a context-dependent and ultimately limited benefit (Gleber-Netto et al., 2026). In contrast, β-adrenergic blockade has been shown to restore T Cell priming, enhance vaccine efficacy, and improve responses to immunotherapy (Daher et al., 2019; Hinkle et al., 2021). Notably, some of these effects are CD4^+^ T cell-dependent, including the induction of cytotoxic Th1 responses and synergy with ICB (e.g., anti-CTLA-4) in metastatic models (Fjæstad et al., 2025).

**FIGURE 1:**
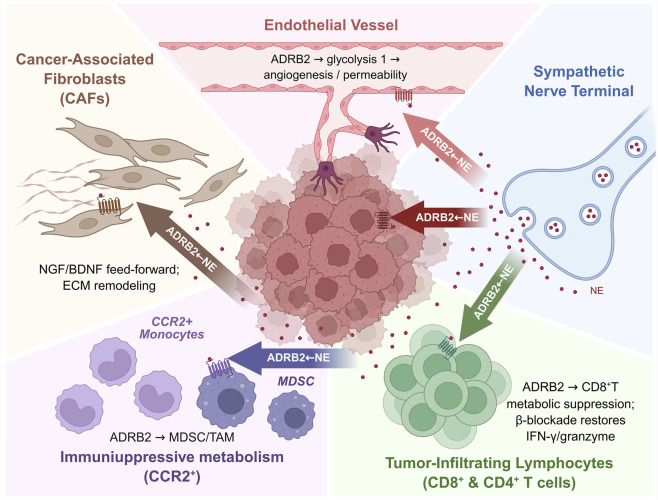
Cell-type-specific ADRB2 signaling in the tumor ecosystem. This schematic summarizes how sympathetic neurotransmission (norepinephrine, NE), derived primarily from sympathetic nerves and, in selected contexts, from local non-neuronal sources, engages β2-adrenergic receptor (ADRB2) on discrete cellular compartments within the tumor microenvironment [tumor cells, cancer-associated fibroblasts (CAFs), vascular endothelium, CCR2^+^ myeloid populations (monocytes/MDSCs/TAMs), and tumor-infiltrating lymphocytes], producing cell type-specific downstream programs that collectively remodel vasculature, extracellular matrix, and antitumor immunity. CAFs and endothelial cells are shown as ADRB2-responsive target cells rather than established NE-producing cells. The diagram also indicates a tumor→neurotrophin (NGF/BDNF) feed-forward loop that promotes intratumoral innervation and amplifies ADRB2-dependent effects. Created with BioRender.com.

Collectively, these findings suggest that ADRB2 signaling broadly suppresses antitumor T Cell immunity, while its therapeutic modulation holds promise for enhancing immunotherapy, with efficacy likely dependent on TME composition, ADRB2 expression patterns, and treatment timing (Figure 1).

#### 3.2.5 Natural killer (NK) cells: impaired cytotoxicity and cytokine secretion

Although current studies rarely provide a direct, detailed account of the specific impact of ADRB2 signaling on NK cell function, within the broader framework of neuro-immune axis regulation, it can be inferred that their function likely undergoes a similar form of inhibition. It is well established that catecholamines released by the SNS broadly modulate the function of immune cells. Within the TME, chronic adrenergic signaling, acting as a component of the immunosuppressive network, is highly likely to impair both the cytotoxicity of NK cells and their capacity to secrete cytokines (such as IFN-γ). This impairment may occur either through direct action on ADRB2 receptors located on NK cells or indirectly by remodeling the microenvironment (e.g., by increasing the levels of inhibitory cytokines) (Zhang F. et al., 2026; Yuan et al., 2025). Such functional compromise further undermines the body’s innate immune surveillance against tumor cells.

## 4 Pharmacological strategies targeting ADRB signaling

Non-selective β-blockers (such as propranolol), serving as classic pharmacological tools targeting adrenergic signaling pathways, have demonstrated anti-tumor potential in various tumor models. Preclinical and clinical studies indicate that propranolol is capable of reversing norepinephrine-mediated pro-tumorigenic effects (Carrasco et al., 2025; Lin et al., 2023; Moisan et al., 2021; O'Logbon et al., 2025). Compared to non-selective blockers, selective ADRB2 antagonists (such as ICI 118,551) offer more precise targeting tools, thereby facilitating a deeper elucidation of the specific roles of ADRB2 signaling within the TME (Rivera-López et al., 2025; Li et al., 2026; Lu et al., 2021). Table 1 summarized pharmacological strategies targeting β-AR signaling and their corresponding translational evidence.

**TABLE 1:** Pharmacological strategies targeting β-AR signaling and their corresponding translational evidence.

Strategy	Mechanism/Targeting	Representative agents/platforms	Advantages	Limitations/Risks	Key evidence and references
Systemic non-selective β-blockade	Block β1/β2 globally (reduces adrenergic tone)	Propranolol (oral)	Readily available; many preclinical/clinical repurposing data; perioperative use feasible	Cardio-respiratory effects, bronchospasm risk; non-specific; mixed epidemiologic results	Preclinical immune + metastasis data; early trials and cohorts (Fjæstad et al., 2025; Ricon-Becker et al., 2023; Wang et al., 2022)
Selective β2 antagonists	Preferential ADRB2 blockade (less cardiac β1 blockade)	Experimental selective β2 antagonists/design	Reduced cardiac toxicity vs. non-selective	Limited clinical agents; off-target effects possible	Mechanistic rationale; preclinical receptor studies (Shin et al., 2026; Pu et al., 2025)
Biased ligands/functional selectivity	Bias signaling away from Gs/cAMP vs. β-arrestin pathways	Research compounds (pharmacology development)	Potential to retain beneficial signaling while avoiding adverse pathways	Early stage; complex pharmacology	Pharmacology literature and GPCR biophysics (Xia et al., 2024)
Cell-targeted delivery (nanoparticles, antibody-drug conjugates, ligand-directed systems)	Deliver antagonists/siRNA to tumor/stroma/myeloid cells	Nanocarriers, ligand-targeted systems, cell-homing platforms	High local concentration; minimize systemic toxicity	Delivery complexity, validation of target specificity	Preclinical NGF siRNA in PDAC; targeted delivery examples (Jin et al., 2026; Shin et al., 2026)
Perioperative short-course blockade (±COX-2)	Time-limited adrenergic suppression around surgery	Propranolol + COX-2 (etodolac) periop regimen	Limits early dissemination/immune suppression; small RCT signal	Requires surgical coordination; heterogenous evidence	COMPIT pilot RCT; biomarker RCTs (Ricon-Becker et al., 2023; Nava et al., 2025)
Neuromodulation/denervation	Surgical or bioelectronic modulation of nerves	Denervation, vagotomy, vagal stimulation	Tissue-local modulation	Irreversible risks (denervation) or device complexity	Denervation studies show tumor effects; context dependent (Lyu et al., 2024; Jin et al., 2026)

Animal studies have demonstrated that β-AR inhibition can convert “cold tumors” into “hot tumors” (which is characterized by enhanced CD8^+^ infiltration and reduced PD-1 expression), and extend survival when combined with chemotherapy or ICB (Renz et al., 2018; Xiong et al., 2024). Epidemiological and retrospective studies regarding propranolol have yielded inconsistent conclusions: while a large pooled cohort study on breast cancer showed no significant survival benefit, smaller-scale studies involving skin cancer and melanoma, along with several clinical reports, suggest potential benefits (Cardwell et al., 2016; Bustamante et al., 2019; Pantziarka et al., 2016). This indicates that tumor type, drug selection, timing of administration, and patient comorbidities are key influencing factors.

## 5 Achieving cell type-specific ADRB2 targeting in experimental and therapeutic context

Cell type-specific targeting of ADRB2 can be operationalized at several complementary levels. First, in experimental settings, causality can be established using compartment-restricted genetic approaches (e.g., Adrb2 conditional loss-of-function in myeloid, endothelial, fibroblast, or T Cell lineages), ideally guided by single-cell and spatial profiling to identify the dominant ADRB2-responsive cellular compartment within a given tumor ecosystem (Zhang et al., 2025; Nguyen et al., 2026). Second, therapeutic specificity can be improved by combining β2-preferring antagonists with cell-directed delivery systems (ligand/antibody-directed nanoparticles or cell-homing carriers) to enrich ADRB2 antagonists or ADRB2/NGF-targeting nucleic acids in selected compartments such as CCR2^+^ monocytes/MDSCs/TAMs, CAF-rich stroma, or tumor vasculature, thereby increasing local exposure while limiting systemic cardiopulmonary toxicity (Kobayashi et al., 2025; Chung et al., 2016; Yang et al., 2023). Third, cell-intrinsic engineering strategies (e.g., ADRB2 pathway inhibition within adoptively transferred T Cells) offer a highly specific way to uncouple adrenergic immunosuppression from whole-body β-blockade (Ajmal et al., 2024). In parallel, time-restricted perioperative regimens provide a pragmatic “temporal specificity” that can suppress adrenergic-driven dissemination and early immunosuppression during a biologically vulnerable window. To operationalize these layered strategies, Table 2 summarizes representative cell type-specific targeting options across major TME compartments. For each compartment, we list example markers or enrichment cues, feasible delivery modalities, and the expected biological endpoints of compartment-selective ADRB2 modulation.

**TABLE 2:** Cell type-specific strategies for ADRB2 targeting in experimental and translational settings.

Cell type	Representative targeting markers/ligands	Delivery modality	Expected endpoints	References
Tumor cells	Tumor-context-specific antigens or ligands; ADRB2 as the functional pharmacologic target	Tumor-homing nanoparticles; ligand-directed carriers; intratumoral delivery of selective ADRB2 antagonists, siRNA, or CRISPR constructs	Inhibit proliferation, EMT, invasion, angiogenesis, and PD-L1 upregulation; reduce metastatic dissemination	Jin et al. (2023), Huang et al. (2021), Xu et al. (2025), Chen et al. (2025), Zhang et al. (2025)
Cancer-associated fibroblasts (CAFs)/stromal fibroblasts	FAP, PDGFRβ, CD146, stromal ECM-associated signatures	CAF-directed nanoparticles or stromal-homing carriers loaded with ADRB2 antagonists or nucleic acids	Suppress CAF activation and ECM remodeling; disrupt perineural invasion niches; improve drug penetration	Liu et al. (2022), Li et al. (2025), Meng W. et al. (2026), Xue et al. (2023), Eng et al. (2014), Hanoun et al. (2015), Yang et al. (2023)
Endothelial cells/vasculature	VEGFR2/KDR, PECAM1/CD31, CDH5	Vascular-targeted antibodies/ligands; endothelial-homing nanoparticles; short-course blockade	Normalize vasculature, reduce permeability and hypoxia, and facilitate immune-cell infiltration	Wang et al. (2025), Kim et al. (2026), Kilpatrick et al. (2019), Lu et al. (2021), Fjæstad et al. (2022)
Myeloid compartment (CCR2^+^ monocytes, MDSCs, TAMs)	CCR2, CD11b, CSF1R, S100A8/9, CD206	Myeloid-homing nanoparticles; CCR2-targeted systems; perioperative propranolol ± COX-2 inhibition	Reduce recruitment and suppressive function; promote antigen presentation; restore Th1/CD8 responses	Rivera-López et al. (2025), Mohammadpour et al. (2021), Shin et al. (2026), Daher et al. (2019), Hinkle et al. (2021), Fjæstad et al. (2022)
T lymphocytes/CAR-T cells	ADRB2, CD3, CD8; activation/exhaustion markers (CD69, CD107a, GZMB, PD-1, TIM3)	*Ex vivo* ADRB2 knockdown or CRISPR editing; pharmacologic blockade during adoptive transfer	Enhance activation, cytotoxicity, metabolic fitness, and persistence; reduce exhaustion	Fjæstad et al. (2025), Gleber-Netto et al. (2026), Pu et al. (2025), Daher et al. (2019), Bucsek et al. (2017), Ajmal et al. (2024)
Natural killer (NK) cells	ADRB2, NKp46, CD56/NK1.1	*Ex vivo* ADRB2 suppression or systemic/local β-blockade	Increase degranulation, IFN-γ production, and cytotoxicity	Meng X. L. et al. (2026), Pu et al. (2025)

## 6 Translational lessons and future directions

Emerging evidence highlights several critical considerations for translating ADRB2-targeted strategies into clinical practice. First, timing appears to be a key determinant of efficacy (Ricon-Becker et al., 2023). Perioperative intervention represents a biologically plausible window to limit tumor cell dissemination and early immunosuppression, while short-course regimens may achieve therapeutic benefit with reduced systemic toxicity. Second, appropriate patient and tumor selection is essential (Kim et al., 2026; Carrasco et al., 2025; Li et al., 2023; Wei et al., 2021; Yang et al., 2022; Jin et al., 2022; Long et al., 2023). Candidate stratification factors include tumor-intrinsic ADRB2 expression, nerve density within the TME, myeloid cell burden, and tumor MHC-II status, *etc.* Advanced approaches such as single-cell and spatial profiling may further refine patient selection by identifying the dominant cellular compartments mediating adrenergic responses (Wang et al., 2025). Third, the incorporation of mechanistic endpoints into clinical trials is crucial (Fjæstad et al., 2025; Xia et al., 2024). Pre-specified biomarkers, including immune cell composition (e.g., CCR2^+^ monocytes, Th1 CD4^+^/CD8^+^ function), circulating cytokine profiles (e.g., CSF1, IL-12, IFNγ), and tumor MHC-II expression, will be essential for linking biological activity to clinical outcomes.

Despite promising advances, several key challenges remain. Establishing causality and cell-type specificity is a major priority, as many current clinical and epidemiological observations are confounded. Definitive cell type-specific ADRB2 targeting requires a layered strategy. In experimental settings, cell type-restricted conditional models, together with single-cell RNA sequencing and spatial transcriptomics, can be used to identify the dominant ADRB2-responsive compartment and validate causality (Zhang et al., 2025; Nguyen et al., 2026). In translational settings, specificity may be improved by combining β2-preferring antagonists or biased ligands with targeted delivery systems (e.g., ligand-directed nanoparticles, antibody-based carriers, or cell-homing nanoplatforms) and, when appropriate, time-limited perioperative blockade (Yang et al., 2023; Ajmal et al., 2024; Fjæstad et al., 2022). Such approaches can be tailored to tumor cells, myeloid cells, stromal fibroblasts, or endothelial cells depending on the intended biological endpoint. At present, these strategies should be viewed as complementary tools for compartment-selective modulation rather than fully established cell type-absolute therapies.

**FIGURE 2:**
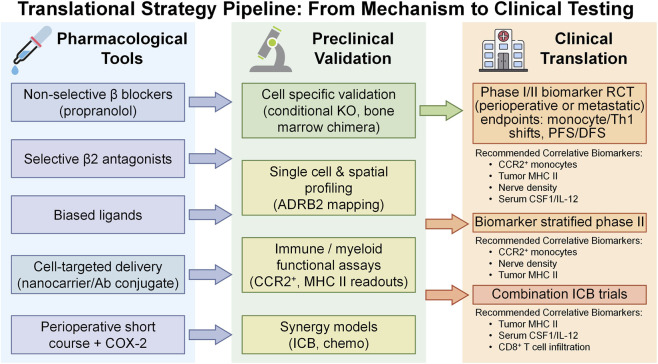
Translational strategy pipeline: from mechanism to clinical testing. Flowchart illustrating how cell type-specific mechanistic evidence for ADRB2-mediated tumor ecosystem remodeling can be translated into pharmacological or engineering interventions and clinical testing. Left column lists intervention modalities (non-selective β-blockers, selective β2 antagonists, biased ligands, cell-targeted delivery, neuromodulation); middle column indicates preclinical validation steps; right column summarizes prioritized early clinical trial designs and mechanistic/biomarker end points.

Definitive evidence will require cell-type-specific conditional models and targeted delivery approaches to delineate the roles of ADRB2 signaling across tumor, myeloid, and endothelial compartments. In addition, the optimal timing and dosing of ADRB2 blockade remain unclear, as perioperative and chronic interventions may exert distinct immunological effects; randomized controlled trials incorporating immune endpoints are needed to address this issue. Safety considerations also warrant attention, as non-selective β-blockers may pose cardiopulmonary risks in vulnerable populations, highlighting the need for more selective agents or localized delivery strategies. Furthermore, robust biomarker development is essential for patient stratification. Integrating single-cell and spatial transcriptomics to assess ADRB2 expression and neural density, alongside composite biomarkers combining ADRB2 levels, CCR2^+^ myeloid signatures, and tumor MHC-II expression, may improve trial design and therapeutic precision. Finally, rational combination strategies represent a high-priority direction, including ADRB2 blockade with myeloid-targeting therapies (e.g., CCR2 inhibitors), ICBs (particularly CTLA-4 blockade), or perioperative anti-inflammatory interventions. Future clinical trials should adopt randomized, biomarker-stratified designs with integrated mechanistic correlative studies (such as analyses of blood, tumor-draining lymph nodes, and tumor biopsies), to capture early immunological changes prior to large-scale outcome evaluation (Figure 2).

## 7 Conclusion

ADRB2 is an integrator of neural, stromal and immune signals in the tumor ecosystem. Cell-type-specific modulation, whether by selective pharmacology, targeted delivery, or timed perioperative intervention, offers a promising route to reshape the TME toward immune permissiveness and reduced metastasis. Translational momentum exists, but rigorous, biomarker-driven randomized studies and cell-targeted approaches are required to establish efficacy and safety across tumor types. It is imperative to conduct an in-depth analysis of the heterogeneity of ADRB2 signaling across different cancer types and individual patients, and to identify biomarkers capable of predicting therapeutic efficacy, in order to guide personalized, stratified treatment strategies. Ultimately, bridging mechanistic insights from bench to bedside will be crucial for realizing the therapeutic promise of these strategies and reshaping clinical management.
